# P-1268. Phage Sensitivity Profiles and their Association with Biofilm-mediated Antibiotic Tolerance in DNS-MRSA Isolates

**DOI:** 10.1093/ofid/ofaf695.1458

**Published:** 2026-01-11

**Authors:** Callan Bleick, Sean R Van Helden, Andrew D Berti, Michael J Rybak

**Affiliations:** Anti-Infective Research Laboratory, College of Pharmacy and Health Sciences, Wayne State University, Detroit, MI, Wayne State University School of Medicine, Department of Microbiology and Immunology, Detroit, MI, Detroit, Michigan; Wayne State University, Eugene Applebaum College of Pharmacy and Health Sciences, Detroit, Michigan; Wayne State University Colleges of Pharmacy and Medicine, Detroit, Michigan; Eugene Applebaum College of Pharmacy and Health Sciences, Detroit, Michigan

## Abstract

**Background:**

Daptomycin non-susceptible MRSA (DNS-MRSA) presents a significant therapeutic challenge due to resistance to multiple antibiotic agents and biofilm-associated antibiotic tolerance. While phage therapy is a promising alternative, resistance mechanisms and strain-specific differences in biofilm formation may hinder efficacy.

**Table 1.**
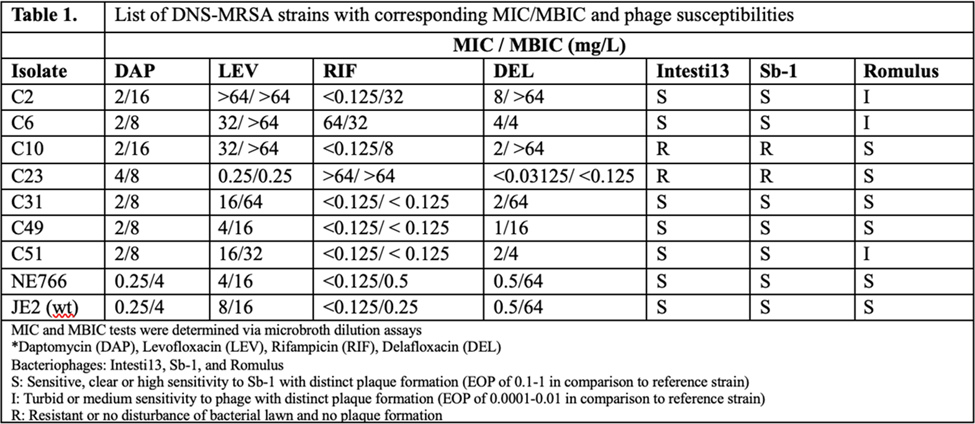
List of DNS-MRSA strains with corresponding MIC/MBIC and phage susceptibilities MIC and MBIC tests were determined via microbroth dilution assays *Daptomycin (DAP), Levofloxacin (LEV), Rifampicin (RIF), Delafloxacin (DEL) Bacteriophages: Intesti13, Sb-1, and Romulus S: Sensitive, clear or high sensitivity to Sb-1 with distinct plaque formation (EOP of 0.1-1 in comparison to reference strain) I: Turbid or medium sensitivity to phage with distinct plaque formation (EOP of 0.0001-0.01 in comparison to reference strain) R: Resistant or no disturbance of bacterial lawn and no plaque formation

**Methods:**

We characterized the antimicrobial susceptibility and biofilm-associated tolerance of several DNS-MRSA clinical isolates and two transposon mutagenized *S. aureus* strains: wild-type JE2 (wt) and NE766 (deficient in poly-N-acetylglucosamine-mediated biofilm production). MIC and MBIC values were determined via broth microdilution for daptomycin (DAP), levofloxacin (LEV), rifampin (RIF), and delafloxacin (DEL). Phage susceptibility was previously determined via plaque assays. Antibiotic and phage resistance mechanisms, including mutations in *gyrA*, *gyrB, qacC,* and *parC* were identified through whole-genome sequencing.

**Results:**

Among the DNS-MRSA isolates, we observed discordance between MIC and MBIC values, particularly for rifampin and delafloxacin, in *Kayvirus* phage-resistant strains C10 and C23. These strains also harbored dual *gyrA* and *parC* mutations. In contrast, NE766 displayed notably lower MBICs across all antibiotics tested (Table 1), despite comparable MICs to JE2 (wt). Strains with susceptibility to all three phages demonstrated uniformly low MBICs, suggesting a potential correlation between phage susceptibility and reduced biofilm-associated tolerance.

**Conclusion:**

These results suggest that phage resistance, particularly to *Kayvirus*-type phages, may serve as a potential marker of biofilm-associated antibiotic tolerance in certain clinical DNS-MRSA strains. While phage susceptibility alone does not guarantee improved antibiotic activity, combined phage-antibiotic profiling and biofilm characterization may offer a more comprehensive approach to identifying recalcitrant phenotypes and tailoring therapy.

**Disclosures:**

Michael J. Rybak, PharmD, PhD, MPH, Abbvie: Grant/Research Support|Innoviva: Grant/Research Support|Melina: Grant/Research Support|Merck: Grant/Research Support|Shionogi: Grant/Research Support

